# Convergent Metabotropic Signaling Pathways Inhibit SK Channels to Promote Synaptic Plasticity in the Hippocampus

**DOI:** 10.1523/JNEUROSCI.1160-18.2018

**Published:** 2018-10-24

**Authors:** Cezar M. Tigaret, Sophie E.L. Chamberlain, Joseph H.L.P. Sadowski, Jeremy Hall, Michael C. Ashby, Jack R. Mellor

**Affiliations:** ^1^Centre for Synaptic Plasticity, School of Physiology, Pharmacology and Neuroscience, University of Bristol, Bristol, BS8 1TD, United Kingdom and; ^2^Neuroscience and Mental Health Research Institute, School of Medicine, Cardiff University, Cardiff, CF24 4HQ, United Kingdom

**Keywords:** hippocampus, metabotropic glutamate receptors, muscarinic receptors, SK channels, spike timing-dependent plasticity, synaptic plasticity

## Abstract

Hebbian synaptic plasticity at hippocampal Schaffer collateral synapses is tightly regulated by postsynaptic small conductance (SK) channels that restrict NMDA receptor activity. SK channels are themselves modulated by G-protein-coupled signaling pathways, but it is not clear under what conditions these are activated to enable synaptic plasticity. Here, we show that muscarinic M1 receptor (M1R) and type 1 metabotropic glutamate receptor (mGluR1) signaling pathways, which are known to inhibit SK channels and thereby disinhibit NMDA receptors, converge to facilitate spine calcium transients during the induction of long-term potentiation (LTP) at hippocampal Schaffer collateral synapses onto CA1 pyramidal neurons of male rats. Furthermore, mGluR1 activation is required for LTP induced by reactivated place-cell firing patterns that occur in sharp-wave ripple events during rest or sleep. In contrast, M1R activation is required for LTP induced by place-cell firing patterns during exploration. Thus, we describe a common mechanism that enables synaptic plasticity during both encoding and consolidation of memories within hippocampal circuits.

**SIGNIFICANCE STATEMENT** Memory ensembles in the hippocampus are formed during active exploration and consolidated during rest or sleep. These two distinct phases each require strengthening of synaptic connections by long-term potentiation (LTP). The neuronal activity patterns in each phase are very different, which makes it hard to map generalized rules for LTP induction onto both formation and consolidation phases. In this study, we show that inhibition of postsynaptic SK channels is a common necessary feature of LTP induction and that SK channel inhibition is achieved by separate but convergent metabotropic signaling pathways. Thus, we reveal a common mechanism for enabling LTP under distinct behavioral conditions.

## Introduction

Spatial representations of the environment are stored within the hippocampus as ensembles of place cells. These ensembles are created during active exploration by Hebbian synaptic plasticity between coactive place cells (Harris et al., 2003; O'Neill et al., 2010). The enhancement of synaptic strength functionally binds place cells together facilitating subsequent reactivation of the entire ensemble (Dragoi et al., 2003; Dupret et al., 2010). These reactivation events occur primarily on a compressed timescale within transient high-frequency oscillatory events termed sharp-wave ripples (SWRs) during resting or sleep states (Wilson and McNaughton, 1994; Lee and Wilson, 2002; Diba and Buzsáki, 2007) and are critical for memory consolidation (Marshall et al., 2006; Girardeau et al., 2009). The process of memory consolidation ultimately leads to the transfer and storage of information in the neocortex but there is also an early stage of ensemble consolidation within the hippocampus which requires synaptic plasticity induced by reactivated patterns of place-cell firing (King et al., 1999; Sadowski et al., 2016). This two-stage model for memory encoding and consolidation within the hippocampus predicts that synaptic plasticity is fundamental to both stages (O'Neill et al., 2010; Atherton et al., 2015), but each stage engages plasticity that is induced by place-cell firing patterns on very different timescales.

Spike timing-dependent plasticity (STDP) rules are commonly used to predict the plasticity outcome from place-cell activity patterns. The original exposition of STDP in the hippocampus showed that precisely timed action potentials in synaptically-coupled neurons determine the direction and magnitude of synaptic plasticity (Bi and Poo, 1998; Debanne et al., 1998). Initial experiments suggested that long-term potentiation (LTP) and long-term depression could be induced by single pairs of presynaptic and postsynaptic spikes but this model has been revised to include a requirement for multiple postsynaptic spikes (Wittenberg and Wang, 2006; Buchanan and Mellor, 2007) and a potentiation only rule for mature hippocampal synapses (Mishra et al., 2016; Tigaret et al., 2016). These stringent rules for LTP induction predict that place-cell activity patterns that occur during both exploration and reactivation are sufficient to induce LTP. However, this is not the case. Instead, place-cell firing patterns during exploration also require activation of muscarinic M1 receptors (M1Rs; Isaac et al., 2009; Buchanan et al., 2010), whereas reactivated place-cell firing patterns require additional coordinated synaptic activity found during SWRs (Sadowski et al., 2016). It is not clear whether a common mechanism underlies these two additional requirements for LTP induction.

Within dendritic spines of CA1 pyramidal cells, NMDA receptors (NMDARs) and small conductance calcium activated potassium (SK/K_Ca2_) channels form a regulatory feedback loop that restricts NMDAR activity, spine Ca^2+^ transients, and therefore LTP induction (Faber et al., 2005; Ngo-Anh et al., 2005; Bloodgood and Sabatini, 2007). Activation of M1Rs, which can also be found in spines (Yamasaki et al., 2010), inhibits SK channels via a Gq-coupled pathway thereby removing the regulation of NMDARs and facilitating spine Ca^2+^ transients and LTP (Buchanan et al., 2010; Giessel and Sabatini, 2010; Dennis et al., 2016). mGluR1 also inhibits SK channels and facilitates spine Ca^2+^ transients and LTP induction at CA1 Schaffer collateral synapses (Tigaret et al., 2016) suggesting a common mechanism of action for mGluR1 and M1R similar to that shown for enhancement of intrinsic membrane properties (Park and Spruston, 2012). During exploration acetylcholine release within the hippocampus is high but during reactivation in SWRs, and particularly SWRs occurring during non-REM sleep, levels of acetylcholine release are very low (Marrosu et al., 1995; Teles-Grilo Ruivo et al., 2017). mGluR1 inhibition of SK channels and facilitation of spine Ca^2+^ transients and LTP induction is favored by glutamate release coupled with strong dendritic depolarization such as might occur during the coordinated synaptic activity found during SWRs. Therefore, we have tested the hypothesis that M1Rs and mGluR1 are active during different behavioral states but act through a common signaling pathway to inhibit SK channels and facilitate LTP induction.

## Materials and Methods

### 

#### 

##### Slice preparation.

Acute transverse hippocampal slices were prepared from adult (P50–P55, 200–250 g) male Wistar rats after a lethal dose of isoflurane inhalation, in accordance with Home Office guidelines as directed by the Home Office Licensing Team at the University of Bristol. Hippocampi were dissected in ice-cold slicing solution containing the following (in mm): 119 NaCl, 10 glucose, 26.2 NaHCO_3_, 2.5 KCl, 1 NaH_2_PO_4_, 0.5 CaCl_2_, and 5 MgCl_2_, and then mounted on agar and cut in 400-μm-thick slices using a VT1200 vibratome (Leica). Slices were incubated in artificial CSF (aCSF) containing the following (in mm): 119 NaCl, 10 glucose, 26.2 NaHCO_3_, 2.5 KCl, 1 NaH_2_PO_4_, 2.5 CaCl_2_, and 1.3 MgCl_2_, at 36°C for 30 min, and then stored at room temperature until use. Solutions were equilibrated with 95% CO_2_ and 5% O_2_ and had osmolarity of 300–310 mOsm. For synaptic plasticity experiments, the slices were cut between CA3 and CA1 before being transferred to the recording chamber.

##### Electrophysiology.

Whole-cell patch-clamp recordings were made from CA1 pyramidal neurons visualized under infrared differential interference contrast (Scientifica SliceScope Pro 6000 or Olympus BX-51 microscope) in a submerged recording chamber superfused with aCSF (∼1.5–2 ml/min) at 35°C containing 50 μm picrotoxin to block GABA_A_ receptors. Patch electrodes (3–5 MΩ) were pulled from borosilicate filamented glass capillaries (Harvard Apparatus) and filled with intracellular solution (in mm): 117 KMeSO_3_, 8 NaCl, 1 MgCl_2_, 10 HEPES, 0.2 EGTA, 4 MgATP, and 0.3 Na_2_GTP, pH 7.2, 280 mOsm.

Recordings were made using a MultiClamp 700A amplifier (Molecular Devices), analog filtered at 4 kHz, and digitized at 10 kHz with a CED Micro 1401 MkII board and Signal 5 acquisition software (Cambridge Electronic Design). Synaptic responses were evoked with tungsten bipolar stimulating electrodes (100 kΩ, 119 μm tip spacing, MicroProbes) or monopolar stimulating electrodes (aCSF-filled patch pipettes) delivering 0.1–1 ms square pulses (Digitimer). Back-propagated postsynaptic action potentials (bAPs) were elicited through pulses of somatic current injections (1–2 nA, 2 ms).

**Figure 1.**
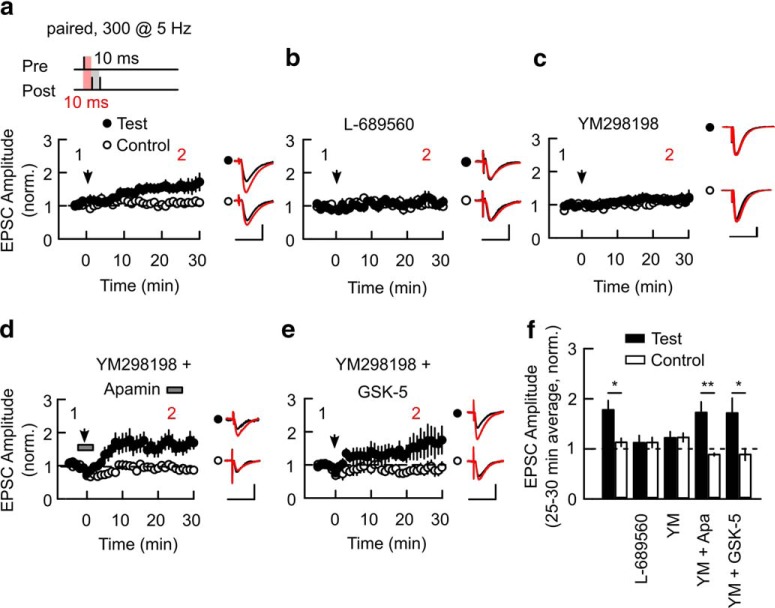
M1R activation or direct block of SK channels rescue LTP after block of mGluR1. ***a***–***c***, Pairing of a single EPSP with two bAPs during a theta frequency train (paired) induces homosynaptic LTP (***a***) that is blocked by the Gly site NMDAR antagonist L-689560 (5 μm; ***b***) or by the mGluR1-selective antagonist YM (0.1 μm; ***c***). ***d***, SK channel blocker apamin (Apa, 0.1 μm) applied during LTP induction (gray bar) rescues LTP from block by YM. ***e***, Coapplication of M1R-selective agonist GSK-5 (1 μm) for the entire experiment rescues LTP from block by YM. Plots in ***a***–***e*** show the time course of EPSC amplitude (mean ± SEM) in Test and Control pathways normalized to the average amplitude 5 min before the paired protocol was delivered to the Test pathway (arrowheads). Insets, Average EPSC waveforms before (1, black) and 25–30 min after LTP induction (2, red). Scale bars: 50 pA, 50 ms. ***f***, Summary of changes in the average EPSC amplitude 25–30 min after LTP induction. *p < 0.05, **p < 0.01. Data shown as mean ± SEM. Stimulus schematic is not drawn to scale.

Synaptic plasticity experiments were conducted by recording synaptic responses in voltage-clamp (−70 mV). Membrane voltage was not corrected for liquid junction potential, which was calculated to be ∼−9 mV. EPSCs (40–60 pA) were evoked at 0.1 Hz alternatively in test and control pathways by stimulating electrodes placed in stratum radiatum on opposite sides of the recorded cell and at different distances from the stratum pyramidale. For the experiments involving SWRs a third pathway was established by placing a stimulating electrode in statum radiatum (see Fig. 5, schematic). All pathways were tested for independence by paired-pulse protocols (Sadowski et al., 2016). The pathways were tuned in current-clamp to evoke subthreshold single or summated EPSPs before baseline recording. Consecutive EPSCs were averaged online every minute and the amplitude was normalized off-line to the average of 3–5 min before the plasticity induction protocol (baseline). Series resistance was monitored throughout the recording and cells with series resistance >30 MΩ or with >20% change in series resistance were discarded. Plasticity induction protocols were applied in current-clamp within 10 min of establishing whole-cell configuration to avoid LTP washout. In all synaptic plasticity experiments, the Test pathway received the conditioning protocol, whereas the Control pathway was left unperturbed. For STDP the conditioning protocol was a theta frequency (5 Hz) train of 300 stimulations for 1 min. Each stimulation consisted of a single EPSP followed by two back-propagated action potentials, all delivered at 100 Hz (“paired” protocol; Fig. 1*a*, schematic). For plasticity induction with place-cell natural spike trains (NSTs) the conditioning was generated by replaying a CA1 spike train as a sequence of somatically-elicited action potentials in the recorded cell (postsynaptic spike train), together with the delivery of a CA3 spike train to the Test pathway (presynaptic spike train). When used, the SWR-associated synaptic input was activated by short trains of five subthreshold EPSPs at 100 Hz delivered to the SWR pathway (Sadowski et al., 2016).

##### Two-photon Ca^2+^ imaging.

**Figure 2.**
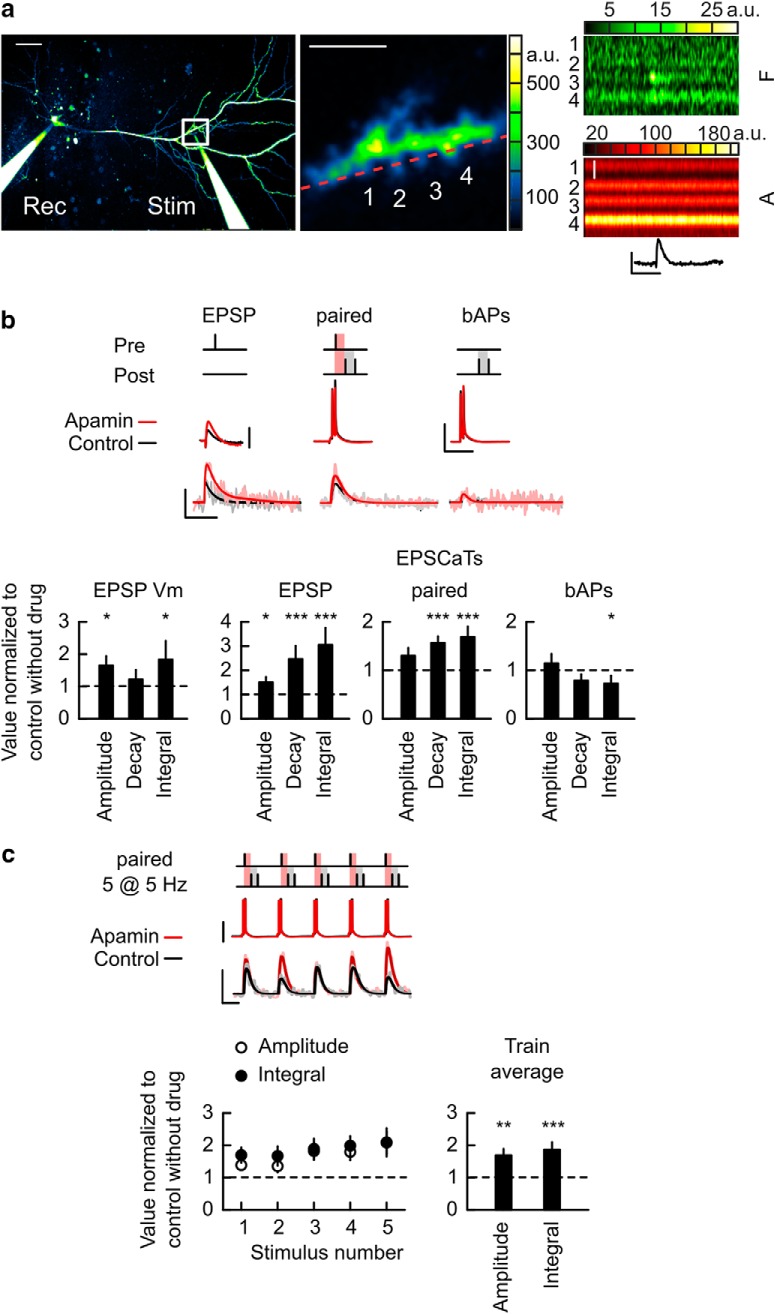
Direct SK channel blockade enhances EPSCaTs. ***a***, Two-photon line-scan imaging of EPSCaTs in CA1 pyramidal neurons. Left, Pseudo-color image (Alexa channel) of a cell patch-loaded with AlexaFluor 594 (***A***) and Fluo 5F (***F***) through the recording electrode (Rec); an Alexa-filled patch electrode (Stim) was placed near an oblique apical dendrite for synaptic stimulation. The white rectangle marks the area of spine imaging, enlarged in the middle. Four spines (middle, numbered) were imaged for EPSCaTs in line-scan mode (red dashed line). Right, Line-scan imaging during an evoked EPSP (top trace) shows synchronized fluorescence transients in the F channel in spines 3 and 4. Image scale bars: left, 50 μm; middle, 5 μm; right (A channel), 2 μm. EPSP scale bars: 5 mV, 0.1 s. ***b***, Effect of apamin on EPSCaTs elicited by individual EPSP, paired, and bAPs stimulations. Top, Examples of somatic *V*_m_ traces and EPSCaTs elicited by the stimulations depicted in the schematics, before (Control, black) and in the presence of 0.1 μm apamin (Apamin, red). Traces are averages from three to four trials. Vertical scale bars: 10 mV (EPSP) and 50 mV; 0.05 ΔF/A (all EPSCaTs). Horizontal scale bars, 0.1 s. Schematics not to scale. Bottom, Summary of changes caused by apamin in EPSP/EPSCaT amplitude (left), decay time constant (middle), and time integral (right) for EPSPs or EPSCaTs evoked by EPSP, paired, and bAPs stimulations. ***c***, Effect of apamin on EPSCaTs elicited during a train of paired stimulations delivered at 5 Hz for 1 s. Top, Train stimulus schematic (not to scale), somatic *V*_m_ traces and EPSCaTs before (Control, black) and during bath application of apamin (apamin, red). Vertical scale bars: 50 mV (membrane potential traces) and 0.05 ΔF/A (EPSCaTs). Horizontal scale bar, 0.1 s. Bottom, Summary of apamin effects on EPSCaT amplitude and time integral for the individual stimuli in the paired train (left) and for the average across the train (right). *p < 0.05, **p < 0.01, ***p < 0.001. Data shown as mean ± SEM. For extended data, see Figure 2-1.

**Figure 3.**
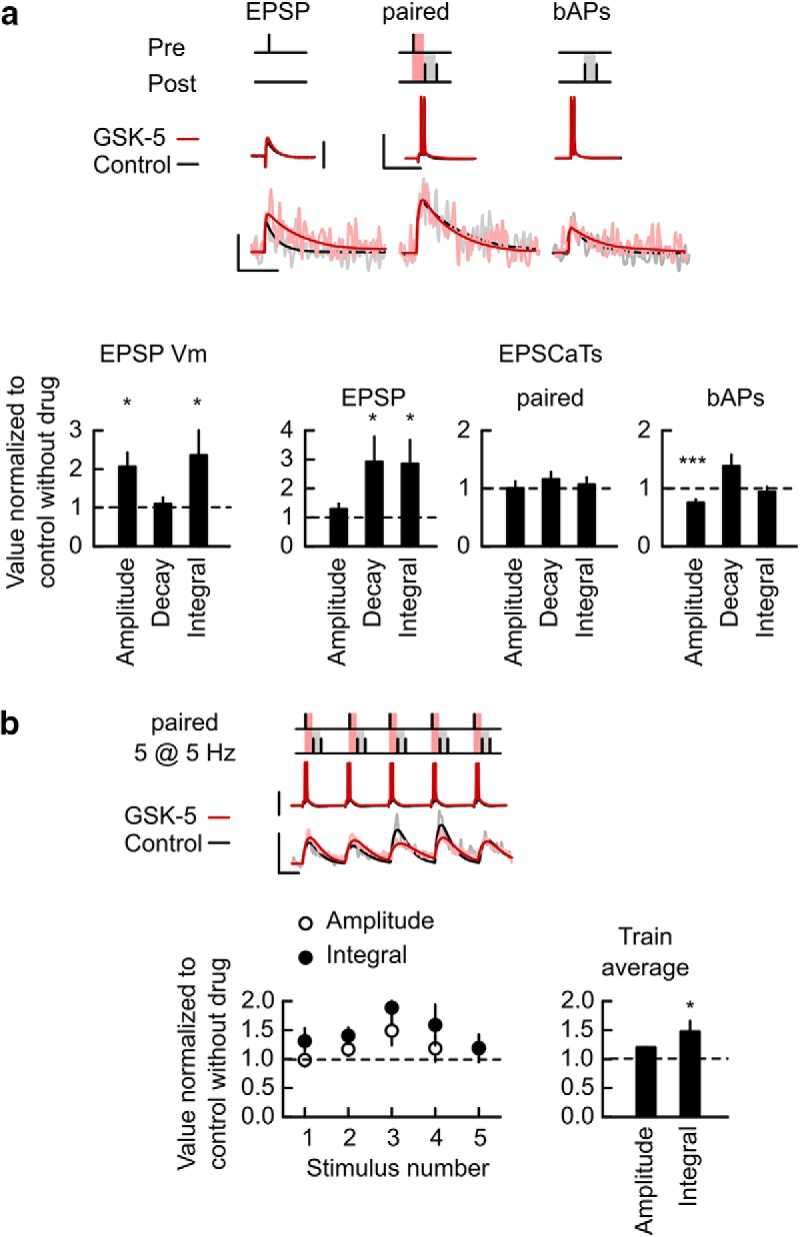
M1R activation provides limited enhancement of EPSCaTs. ***a***, Effect of GSK-5 on EPSCaTs elicited by EPSP, paired and bAPs stimulations. Top, Example traces for somatic *V*_m_ and spine EPSCaT elicited by the stimulations depicted in the schematics, before (Control, black) and in the presence of 1 μm GSK-5 (red). Vertical scale bars: 10 mV (1Pre) and 50 mV; 0.05 ΔF/A (all EPSCaTs). Horizontal scale bars, 0.1 s. Traces are averages of three to four trials. Bottom, Summary of changes caused by GSK-5 in EPSP/EPSCaT amplitude (left), decay time constant (middle), and time integral (right) for EPSPs or EPSCaTs evoked by EPSP, paired, and bAPs stimulations. ***b***, Effect of GSK-5 on EPSCaTs elicited during a train of paired stimuli at 5 Hz for 1 s. Top, Train stimulus schematic, somatic *V*_m_ and spine EPSCaT traces before (Control, black) and during bath application of GSK-5 (red). Vertical scale bars: 50 mV (membrane potential traces) and 0.05 ΔF/A (EPSCaTs). Horizontal scale bar, 0.1 s. Bottom, Summary of the effect of GSK-5 on individual EPSCaTs amplitude and time integral during the stimulus train (left) and on average EPSCaT across the train (Train average; right). *p < 0.05, ***p < 0.001. Data shown as mean ± SEM. Stimulus schematics are not drawn to scale. For extended data, see Figure 3-1.

**Figure 4.**
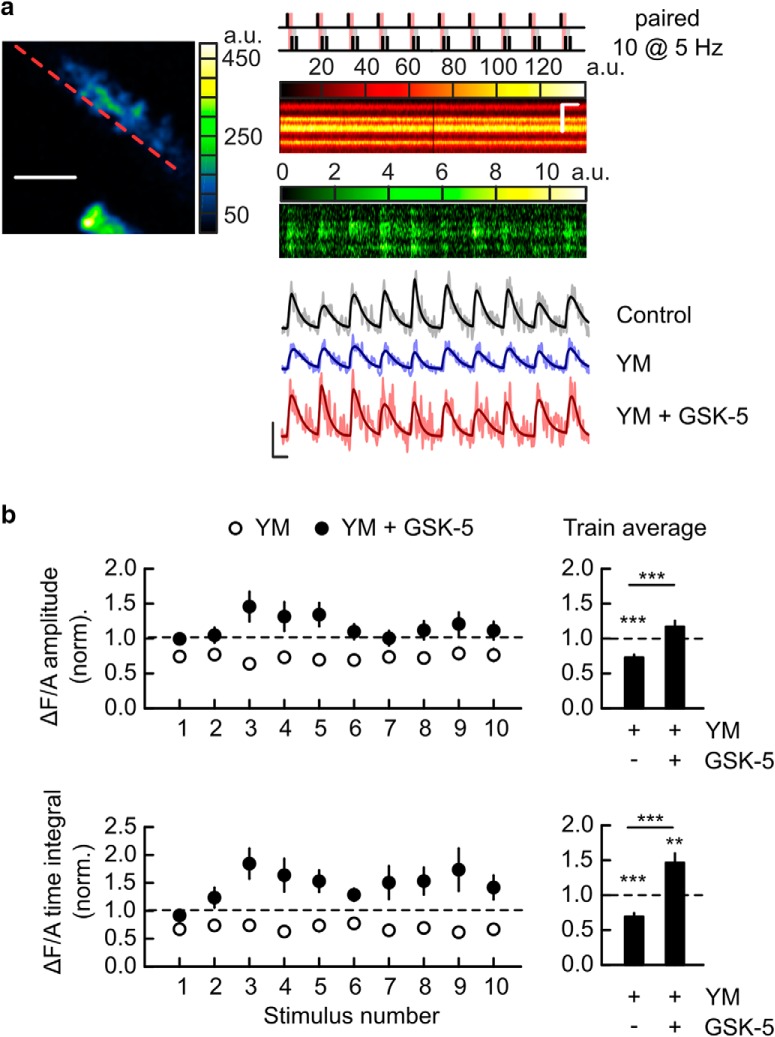
M1R activation restores EPSCaT magnitude during LTP induction when mGluR1 are blocked. ***a***, Example two-photon imaging of EPSCaTs during paired stimulus train (5 Hz, for 2 s; top right schematic). Four spines on the same dendritic segment are shown on the left (Alexa channel) with the line scan overlaid (red dashed line). Right, Fluorescence signal during one line, recorded in Alexa channel (middle) and Fluo-5F channel (bottom) across the four spines shown on left, during the application of the stimulus train. Scale bars: horizontal, 5 μm (left) and 0.2 s (right); vertical (right), 2 μm. ***b***, Top, Example traces of EPSCaTs elicited by a two second paired stimulus train before (Control, black), during bath application of YM298198 alone (YM, blue) and with YM298198 and GSK-5 together (YM + GSK-5, red). Traces are averages of four to five trials for each condition, recorded at the same spine. Scale bars: 0.05 ΔF/A and 0.1 s. Bottom, Summary of the amplitude (top) and time integral (bottom) for the individual EPSCaTs within the train (left) and for the average EPSCaTs within the train (right) before (Control, gray) and after wash-in of YM (blue) and YM + GSK-5 (red). **p < 0.01, ***p < 0.001. Data shown as mean ± SEM. Schematic not drawn to scale. For extended data, see Figure 4-1.

Spine Ca^2+^ imaging was performed on a Scientifica Multiphoton Imaging System based on a SliceScope Pro 6000. Patch electrodes were filled with intracellular solution containing the following (in mm): 117 KMeSO_3_, 8 NaCl, 1 MgCl_2_, 10 HEPES, 4 MgATP, and 0.3 Na_2_GTP, pH 7.2, 280 mOsm freshly supplemented with the medium affinity fluorescent Ca^2+^ indicator Fluo-5F (200 μm; Life Technologies) and a reference fluorescent dye (Alexa Fluor 594, 30 μm; Life Technologies). EGTA was omitted from the intracellular solution to avoid additional Ca^2+^ buffering capacity being introduced in the cell. Spine Ca^2+^ transients (EPSCaTs) were imaged on secondary radial oblique dendrites of CA1 pyramidal neurons in dual fluorescence (Tigaret et al., 2013) with a 60× water-immersion objective. Fluorescence was excited with a Ti:sapphire laser (Newport Spectra-Physics) tuned to 810 nm. After whole-cell configuration was established in voltage-clamp, cells were switched to current-clamp and subthreshold EPSPs were evoked at 0.1 Hz with a monopolar patch electrode containing aCSF and AlexaFluor 594 (5 μm) for visualization (Fig. 2*a*). The tip of the electrode was placed in stratum radiatum and advanced slowly in the proximity of the imaged dendrite. Cells were dye-loaded for 10–15 min by injecting 100–150 pA inward current before optically responsive spines were detected (Tigaret et al., 2013). EPSCaTs were recorded in line-scanning mode in series of 1000 lines per second for 1 s. Line scans were acquired in batches of up to six repeats every 15–20 s to minimize photodamage (Tigaret et al., 2016). Spines were monitored for drifting in the Alexa channel between batches and drifts <1 μm were compensated. Different stimulation protocols (Figs. 2*a*, 3*a*; EPSP, APs, paired) were interleaved in the same batch. EPSCaT traces were grouped by stimulation type and averaged off-line. For short theta train EPSCaT recordings, paired stimulations were delivered at 5 Hz for the duration indicated in Figures 2*b*, 3*b*, and 4. When drugs were used, Control data were obtained for 5 min prior drug application. Resting membrane potential was continuously monitored. Cells were discarded from the study in any of the following condition: resting membrane potential was >−60 mV or changed by >10 mV; the imaged spines or parent dendrite had localized swelling, sustained increase in resting Ca^2+^ fluorescence or when stimulation induced a tonic increase in Ca^2+^ fluorescence without return to levels before stimulus.

Fluorescence images (12 bit quantization) were acquired with a data acquisition board (National Instruments) driven by ScanImage r3.8 software. Fluorescence data were analyzed off-line with software written in MATLAB (MathWorks). EPSCaT traces were calculated from denoised fluorescence images as the relative change in Fluo-5F versus Alexa fluorescence intensities (ΔF/A) and fitted with an exponential rise and decay exponential function (Tigaret et al., 2013, 2016). The peak ΔF/A amplitude, decay time constant and time integral were derived from the fitted curves. Theta train EPSCaTs were fitted with the sum of 5 or 10 exponential rise and decay curves (for 1 or 2 s trains, respectively) separated by 0.2 s. The amplitude and time integral values were calculated for individual EPSCaTs in the train and for the waveform average across the train. Data were not calibrated for Ca^2+^ concentration. Linearity of EPSCaT summation for single paired stimulation was calculated as the ration between the paired EPSCaT amplitude and the sum of EPSP and bAP-elicited EPSCaTs for each spine. The distances from the imaged spines to the soma was determined *post hoc* using Simple Neurite Tracer (Image/J software; Longair et al., 2011) in three-dimensional reconstructions from *Z*-stacks collected in the AlexaFluor 594 channel at the end of the experiment.

##### Statistical analysis.

Data distributions were tested for normality using Kolmogorov–Smirnov test. For drug applications, tests of significance for EPSCaT responses during drug applications normalized to control before drug were tested using two-sided Wilcoxon rank sum tests. Un-normalized data are shown in extended data Figures 2-1, 3-1, and 4-1. Sample sizes were determined by power calculations based on typical effect sizes. For synaptic plasticity experiments statistical comparisons were made between Test and Control pathways on the baseline-normalized EPSC amplitudes averaged over last 5 min of recording against the null hypothesis of no difference between sample means in both pathways (paired *t* test assuming non-equal variances). The level of significance for all statistical comparisons was set at 0.05. The calculated probabilities are given in Results. Summary data are presented as mean ± SEM. Sample sizes are given in cells for LTP experiments (maximum 3 cells per animal, 1 cell per slice) and as spines and cells for EPSCaT imaging (maximum 2 cells per animal, 1 cell per slice).

## Results

### Modulation of SK channels and facilitation of LTP

Inhibition of SK channels facilitates the induction of spike timing-dependent LTP (STD-LTP) at mature Schaffer collateral synapses onto CA1 pyramidal cells and activation of mGluR1s or M1Rs located on dendritic spines can inhibit SK channels (Ngo-Anh et al., 2005; Buchanan et al., 2010; Tigaret et al., 2016). However, it is not clear that the two receptors can compensate for one another through the common mechanism of SK channel inhibition. Therefore, we tested the dependence of STD-LTP induction on the inhibition of SK channels by three separate mechanisms: direct inhibition of SK channels with apamin or indirect inhibition of SK channels by either mGluR1 or M1R activation. Endogenous release of glutamate during STD-LTP induction is sufficient to activate mGluR1 and therefore to test its role we used a selective mGluR1 antagonist YM298198 (YM; Tigaret et al., 2016). STD-LTP was induced by a train of paired stimuli consisting of 300 synaptic stimulations paired with two postsynaptic action potentials at interstimulus intervals of 10 ms (Fig. 1*a*; see Materials and Methods; Tigaret et al., 2016) delivered at theta frequency (5 Hz). This pairing protocol induced reliable, slowly-developing homosynaptic LTP (Fig. 1*a*,*f*; Test vs Control: 1.59 ± 0.16 vs 1.22 ± 0.07, *p* = 0.029, *n* = 14 cells, 10 animals) similar to that observed previously for STD-LTP and reflecting the lack of an initial post-tetanic potentiation (Pike et al., 1999; Buchanan and Mellor, 2007; Isaac et al., 2009; Tigaret et al., 2016). STD-LTP was NMDAR-dependent because it was blocked by application of the NMDAR antagonist L-689560 (5 μm; Fig. 1*b*,*f*; Test vs Control: 1.1 ± 0.14 vs 1.05 ± 0.09, *p* = 0.18, *n* = 7 cells, 6 animals). STD-LTP also required mGluR1 activation since it was blocked by the mGluR1-selective antagonist YM298198 (0.1 μm; Fig. 1*c*,*f*; Test vs Control: 1.18 ± 0.13 vs 1.12 ± 0.06, *p* = 0.23, *n* = 10 cells, 6 animals).

If there is a common mechanism for facilitating LTP then direct or indirect inhibition of SK channels is predicted to rescue STD-LTP when mGluR1-mediated inhibition of SK channels is blocked. Direct inhibition of SK channels with apamin (0.1 μm) during the STD-LTP induction in combination with continuous application of the mGluR1 antagonist rescued STD-LTP (Fig. 1*d*,*f*; Test vs Control: 1.62 ± 0.19 vs 0.88 ± 0.03, *p* = 0.0014, *n* = 8 cells, 6 animals). Indirect inhibition of SK channels with the selective M1R agonist GSK-5 (Dennis et al., 2016) also rescued STD-LTP in the presence of the mGluR1 antagonist (Fig. 1*e*,*f*; Test vs Control: 1.76 ± 0.38 vs 0.89 ± 0.12, *p* = 0.037, *n* = 10 cells, 5 animals). Importantly, apamin did not change presynaptic function as measured by the paired-pulse ratio (2.26 ± 0.26 control vs 2.69 ± 0.3 apamin, *p* = 0.69, *n* = 18) and we have previously shown that neither GSK-5 nor YM298198 alter presynaptic function (Dennis et al., 2011, 2016; Tigaret et al., 2016). These results indicate that inhibition of SK channels is a common mechanism by which two classes of G-protein-coupled receptors, M1R and mGluR1, facilitate induction of Hebbian LTP.

### Blockade of SK channels facilitates EPSCaTs

EPSCaTs are a key trigger for induction of Hebbian plasticity and are negatively regulated by Ca^2+^-sensitive postsynaptic SK channels (Faber et al., 2005; Ngo-Anh et al., 2005; Bloodgood and Sabatini, 2007; Giessel and Sabatini, 2010; Griffith et al., 2016). Here we tested the effect of the SK channel blocker apamin on EPSCaTs elicited by STD-LTP inducing stimulations. We imaged EPSCaTs on spines located on secondary and tertiary radial oblique dendritic branches of CA1 pyramidal neurons (*n* = 22 spines, 8 cells, 6 animals) at a distance of 271 ± 21 μm from the soma. EPSCaTs were evoked by single subthreshold synaptic stimulation (EPSP), somatically induced pairs of bAPs at 100 Hz, or a combination of both (paired; see Materials and Methods; Fig. 2*a*,*b*, and Fig. 2-1). EPSCaT imaging was performed separately from the LTP experiments to avoid potential LTP washout during dye loading of the cells in whole-cell mode and used the same physiological conditions. We interleaved different stimulation protocols at a low frequency (0.05–0.1 Hz) at the same synapse to directly compare responses while minimizing photodamage. Spine Ca^2+^ transients were detected on 2–3 spines in each dendritic segment imaged (average dendritic segment length: 16.9 ± 2.3 μm) and although they were time-locked with the stimulation, failures in EPSCaT responses were often observed while EPSCaTs were never seen in dendritic shafts (Tigaret et al., 2016). EPSP stimulations elicited EPSCaTs with average ΔF/A amplitude, decay time constant and ΔF/A time integral of 0.026 ± 0.004, 0.063 ± 0.007 s, and (2.1 ± 0.4) × 10^−3^ s, respectively. EPSP EPSCaTs were synchronous with evoked EPSPs recorded at the soma, which had an average amplitude, decay time constant, and time integral of 6.77 ± 0.99 mV, 0.038 ± 0.006 s, and 0.249 ± 0.051 mV·s, respectively. bAPs also evoked EPSCaTs with average ΔF/A amplitude, decay, and time integrals of 0.0199 ± 0.002, 0.052 ± 0.006 s, and (1.74 ± 0.3) × 10^−3^ s, respectively. Paired stimulations evoked EPSCaTs with ΔF/A amplitude, decay, and time integrals of 0.043 ± 0.003, 0.063 ± 0.004 s, and (4.02 ± 0.32) × 10^−3^ s, respectively. Paired EPSCaT amplitudes were a linear summation of the EPSCaTs elicited individually by EPSP and bAPs (linearity factor: 1.12 ± 0.14; Tigaret et al., 2016).

10.1523/JNEUROSCI.1160-18.2018.f2-1 Figure 2-1 **Direct SK channel blockade enhances EPSCaTs.** Data from Figure 2 are replotted without normalization to show values in control and in the presence of apamin. ΔF/A Amplitude and Time Integral linearity factors are also included in b. Download Figure 2-1, TIF file

The SK channel blocker apamin (100 nm) increased EPSP amplitude and time integral (Fig. 2*b* and Fig. 2-1; amplitude: 1.65 ± 0.29, *p* = 0.023; decay: 1.21 ± 0.29, *p* = 0.64; time integral: 1.83 ± 0.58, *p* = 0.039, values normalized to control without drugs; Faber et al., 2005; Ngo-Anh et al., 2005; Bloodgood and Sabatini, 2007) and also increased the amplitude, duration and time integral for EPSCaTs elicited by EPSP or paired stimulations (Fig. 2*b* and Fig. 2-1; EPSP: ΔF/A amplitude 1.51 ± 0.21, *p* = 0.23, decay 2.39 ± 0.52, *p* = 0.0005, and ΔF/A time integral 2.97 ± 0.67, *p* = 0.0005; Paired: amplitude 1.31 ± 0.14, *p* = 0.058, decay 1.57 ± 0.13, *p* = 9.05 × 10^−6^ and time integral 1.72 ± 0.2, *p* = 0.004; *p* values from Wilcoxon rank sum tests, values normalized to control without drugs). In contrast, apamin produced little or no change in EPSCaTs elicited by bAPs (Fig. 2*b* and Fig. 2-1; ΔF/A amplitude 1.12 ± 0.18, *p* = 0.54, decay 0.75 ± 0.12, *p* = 0.07, and ΔF/A time integral 0.67 ± 0.16, *p* = 0.013, values normalized to control without drugs). EPSCaTs evoked by single paired stimulations remained a linear summation of EPSCaTs evoked by EPSP and AP in the presence of apamin (control: 1.11 ± 0.13, apamin: 1.28 ± 0.13, *p* = 0.46).

In support of the idea that LTP induction relies on the generation of large magnitude EPSCaTs, blockade of synaptic mGluR1 results in an inhibition of both STD-LTP and the EPSCaTs evoked during induction (Tigaret et al., 2016). To test whether SK channels also regulate EPSCaTs elicited during STD-LTP induction we performed separate experiments to evoke EPSCaTs during 1 s trains of paired stimulations at 5 Hz in the absence and presence of apamin (Fig. 2*c* and Fig. 2-1). The induction train elicited average EPSCaTs with ΔF/A amplitude and time integral of 0.038 ± 0.0058 and (2.85 ± 0.52) × 10^−3^ s, respectively (Fig. 2*c* and Fig. 2-1). The train EPSCaTs were enhanced in the presence of apamin (ΔF/A amplitude: 1.69 ± 0.2, *p* = 0.002; time integral: 1.86 ± 0.23, *p* = 0.00037, *n* = 24 spines, 7 cells, 3 animals, values normalized to control without drugs), confirming the hypothesis that SK channels are activated and negatively modulate EPSCaTs during STD-LTP induction.

### Effect of M1R agonist GSK-5 on EPSCaTs

Our results suggest that the inhibition of postsynaptic SK channels either directly, or as a consequence of synaptic mGluR1 activation, allows STD-LTP induction through a relief of EPSCaTs from negative SK channel-dependent regulation of NMDARs. Both SK channel inhibition and the rescue of STD-LTP in the absence of mGluR1 signaling can also be achieved by M1R activation. Therefore, we next tested whether EPSCaTs are also enhanced by M1R activation. In contrast to the effects of apamin, GSK-5 (1 μm; Dennis et al., 2016) had much smaller and somewhat inconsistent effects. EPSP amplitudes but not durations were increased (Fig. 3*a* and Fig. 3-1; amplitude 2.06 ± 0.36, *p* = 0.046; decay 1.1 ± 0.16, *p* = 0.57; time integral 2.36 ± 0.7, *p* = 0.015; *n* = 7 cells, 4 animals, values normalized to control without drugs). EPSCaTs evoked by EPSPs had increased durations but not amplitudes, whereas GSK-5 had limited effects on paired or bAP-evoked EPSCaTs (Fig. 3*a* and Fig. 3-1; Paired: amplitude 1.29 ± 0.18, *p* = 0.28; decay 2.93 ± 0.85, *p* = 0.015; time integral 2.86 ± 0.81, *p* = 0.02; *n* = 26 spines, 7 cells, 4 animals, values normalized to control without drugs). The amplitude of EPSCaTs evoked by single paired simulations was a linear summation of the EPSCaTs evoked by EPSP and AP, both during control and after GSK-5 wash-in (control: 0.96 ± 0.06, GSK-5: 0.98 ± 0.07, *p* = 0.81).

10.1523/JNEUROSCI.1160-18.2018.f3-1 Figure 3-1 **M1R activation provides limited enhancement of EPSCaTs.** Data from Figure 3 are replotted without normalization to show values in control and in the presence of GSK-5. ΔF/A Amplitude and Time Integral linearity factors are also included in a. Download Figure 3-1, TIF file

To test the effect of GSK-5 on EPSCaTs elicited during STD-LTP induction trains we performed experiments similar to those described for direct SK channel blockade. These revealed that again GSK-5 had a much smaller effect on EPSCaTs than apamin (Fig. 3*b* and Fig. 3-1; ΔF/A amplitude: 1.2 ± 0.14, *p* = 0.62; time integral: 1.47 ± 0.18, *p* = 0.01; *n* = 20 spines, 5 cells, 3 animals, values normalized to control without drugs).

### M1R activation can restore EPSCaTs when mGluR1 is blocked

The small and inconsistent effects of GSK-5 on EPSPs and EPSCaTs are in contrast to the facilitatory effect of M1R activation on EPSPs (Dennis et al., 2016) and spine Ca^2+^ transients evoked by glutamate uncaging (Giessel and Sabatini, 2010). Because GSK-5 rescues STD-LTP when mGluR1 are blocked (Fig. 1*e*,*f*), whereas mGluR1 activity during paired stimulation facilitates EPSCaTs (Tigaret et al., 2016) we hypothesized that the endogenous synaptic mGluR1 activation during STD-LTP induction may occlude the effects of M1R activation on EPSCaTs. We tested this hypothesis by measuring the effect of mGluR1 antagonist YM298198 (1 μm) alone and coapplied with M1R agonist GSK-5 (1 μm) on EPSCaTs evoked during 2 s STD-LTP induction trains (10 paired stimulations at 5 Hz; Fig. 4). Blockade of mGluR1 reduced EPSCaT magnitudes during the paired stimulation train (Fig. 4*a*,*b* and Fig. 4-1; ΔF/A amplitude and time integral, respectively, for YM: 0.72 ± 0.04, *p* = 1.14 × 10^−7^ and 0.69 ± 0.05, *p* = 1.41 × 10^−6^, values normalized to control without drugs; *n* = 39 spines, 13 cells, 10 animals). EPSCaTs were completely rescued by GSK-5 (Fig. 4*a*,*b* and Fig. 4-1; values normalized to control: ΔF/A amplitude 1.17 ± 0.08, *p* = 0.15 and *p* = 6.85 × 10^−6^ compared with YM alone; time integral 1.46 ± 0.13, *p* = 0.0027 and *p* = 2.32 × 10^−7^ compared with YM alone). These results indicate that during a train of paired stimulations the synaptic activation of mGluR1 is sufficient to inhibit SK channels and facilitate EPSCaTs and to largely occlude further enhancement of Ca^2+^ signals through activation of M1Rs.

10.1523/JNEUROSCI.1160-18.2018.f4-1 Figure 4-1 **M1R activation restores EPSCaT magnitude during LTP induction when mGluR1 are blocked.** Data from Figure 4 are replotted without normalization to show values in control and in the presence of YM298198 or YM298198 + GSK-5. Download Figure 4-1, TIF file

### mGluR1 or M1R activation facilitate the induction of LTP by place-cell NSTs

**Figure 5.**
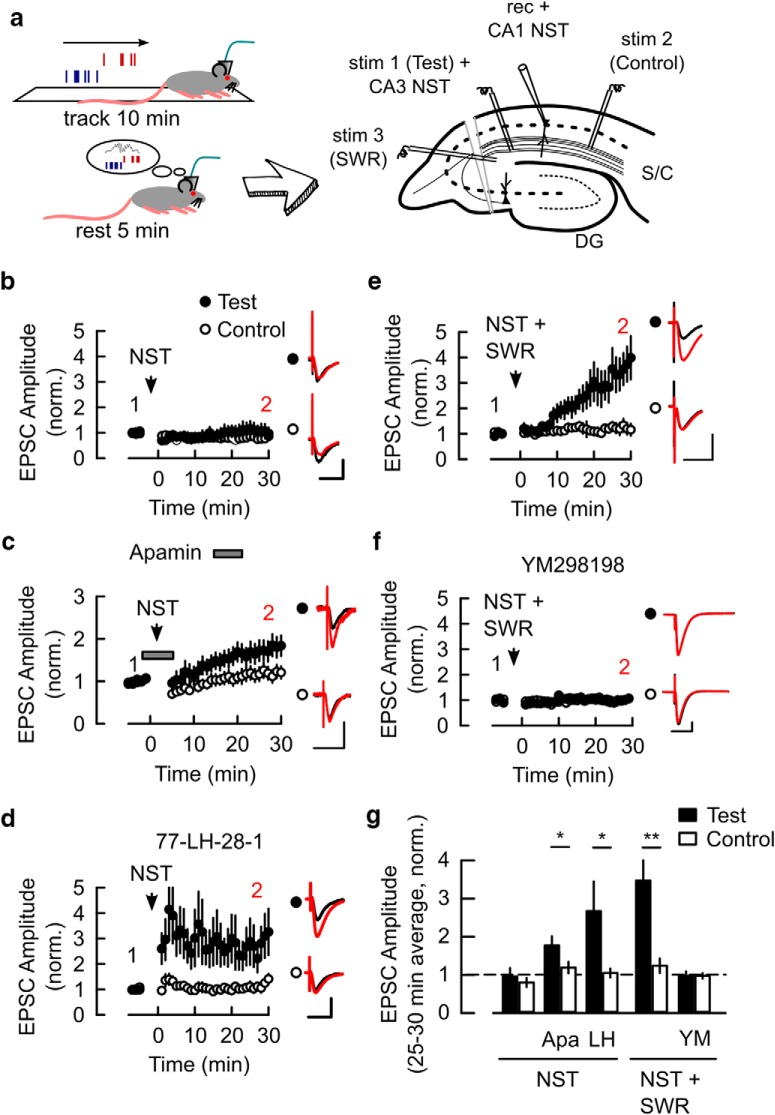
Induction of synaptic plasticity by patterns of reactivated place-cell firing. ***a***, Left, Reactivated place-cell firing patterns and associated SWR timing were recorded *in vivo* from CA3 and CA1 fields during rest (bottom left). The pattern of CA1 place-cell activity was replayed into the recorded CA1 cell (rec + CA1 NST), the pattern of CA3 place-cell activity was replayed into the test pathway (CA3 NST) and the artificial SWR stimulation was given to another input pathway (SWR) when required in LTP experiments in *ex vivo* slices (right; see Materials and Methods). Schematic modified after (Sadowski et al., 2016). ***b***, Application of the NST spike train combination alone did not induce LTP. ***c***, NST stimulation in the presence of Apamin (apa; 0.1 μm) induced pathway-specific LTP. ***d***, NST stimulation in the presence of 77-LH-28–1 (LH; 1 μm) induced pathway-specific LTP. ***e***, NST stimulation in association with artificial SWR stimulation (NST + SWR) induced pathway-specific LTP. ***f***, LTP induced by NST + SWR was blocked in the presence of YM298198 (YM; 1 μm). ***b***–***f***, Plots of the time course of EPSC amplitude in Test and Control pathways normalized to the average amplitude 5 min before NST or NST + SWR were applied (arrowheads). Insets, The average EPSC waveforms before (1, black) and 25–30 min after NST stimulation (2, red). Scale bars: 50 pA and 50 ms. ***g***, Summary of changes in the average EPSC amplitude in the Test and Control pathways 25–30 min after NST stimulation for the conditions in ***b***–***f***. *p < 0.05, **p < 0.01. Data shown as mean ± SEM.

Synaptic plasticity between hippocampal place cells can be induced by natural spike firing patterns or NSTs during active exploration provided there is an appropriate level of cholinergic tone (Isaac et al., 2009) or by NST during restful states in the absence of cholinergic signaling provided that the NST is associated with SWRs (Sadowski et al., 2016). On the other hand, our STD-LTP results indicate a seemingly redundant mechanism whereby two different metabotropic receptors, M1R and mGluR1, converge onto a common signaling pathway to facilitate LTP. We hypothesized that this pathway is also activated during synaptic plasticity induction by NST+SWR. First, we sought to replicate the previous findings whereby the association of NST patterns with SWRs recorded *in vivo* induce LTP. The schematic in Figure 5*a* depicts the experiments where NST patterns recorded from pairs of place cells and SWR patterns recorded on the local field potential (Sadowski et al., 2016) were replayed in *ex vivo* slices. As previously reported (Sadowski et al., 2016), replay of NST alone did not induce LTP (Fig. 5*b*,*g*; Test vs Control pathway: 0.96 ± 0.22 vs 0.79 ± 0.12, *p* = 0.3, *n* = 9 cells, 6 animals). However, NST induced a robust pathway-specific LTP when apamin or the M1R agonist 77-LH-28–1 (1 μm; Buchanan et al., 2010) were present during the experiment (Fig. 5*c*,*d*,*g*; apamin Test vs Control: 1.77 ± 0.24 vs 1.19 ± 0.15, *p* = 0.011, *n* = 10 cells, 4 animals; 77-LH-28-1 Test vs Control: 2.91 ± 0.76 vs 1.17 ± 0.18, *p* = 0.039, *n* = 7 cells, 4 animals), indicating that the M1R signaling pathway facilitates LTP induction by NST under these conditions by inhibition of SK channels. Association of NST with SWR also induced a strong pathway-specific LTP (Fig. 5*e*,*g*; Test vs Control: 3.47 ± 0.64 vs 1.24 ± 0.19 *p* = 0.004, *n* = 9 cells, 6 animals) but this LTP was blocked in the presence of mGluR1 antagonist (Fig. 5*f*,*g*; Test vs Control: 0.97 ± 0.12 vs 0.97 ± 0.07 *p* = 0.98, *n* = 5 cells, 5 animals). These data indicate that NST alone were unable to activate mGluR1 which is not surprising given the highly coordinated but sparse firing with limited postsynaptic burst firing observed during the NST (Sadowski et al., 2016).

These results show that LTP induction by natural spike firing patterns in *ex vivo* slices requires a facilitatory mechanism that can be activated by either M1R or by synaptic activation of mGluR1, with the latter being driven by synaptic activity during the SWR. Given the generally low levels of acetylcholine present during SWR activity (Vandecasteele et al., 2014; Teles-Grilo Ruivo et al., 2017), this indicates that the mechanism of LTP facilitation during replayed place-cell activity patterns is via activation of mGluR1.

## Discussion

**Figure 6.**
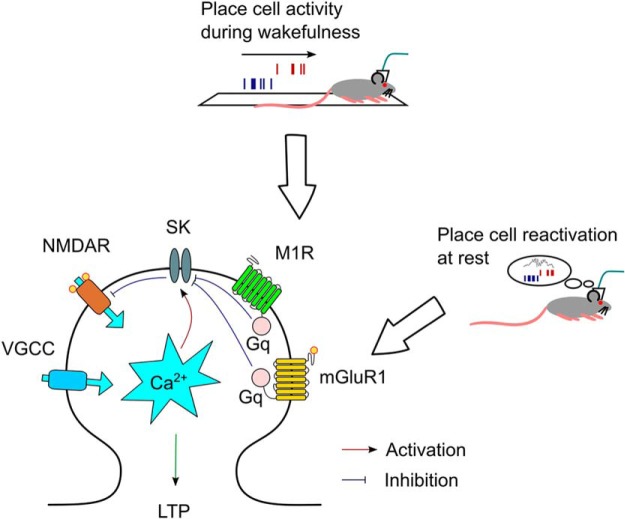
Separate mechanisms for the gating of LTP by inhibition of SK channels during wakefulness or rest. During active exploration, acetylcholine release activates M1Rs that inhibit SK channels and relieve the negative regulation of NMDARs, which results in the induction of LTP between synaptically coupled place cells. During rest, when acetylcholine release is low, coordinated glutamate release during SWRs activates mGluR1 that inhibit SK channels and relieve the negative regulation of NMDARs, which results in further LTP between coactive place cells.

Large Ca^2+^ transients in dendritic spines mediated primarily by NMDARs are required for the induction of synaptic plasticity but these Ca^2+^ signals are tightly regulated by Ca^2+^ activated SK channels located within the spines that hyperpolarize the membrane and act as a negative feedback mechanism on spine excitability (Faber et al., 2005; Ngo-Anh et al., 2005; Bloodgood and Sabatini, 2007; Griffith et al., 2016). Therefore, these dendritically located SK channels act as an effective gate on synaptic plasticity and their activity must be reduced or bypassed before plasticity can be induced (Buchanan et al., 2010; Tigaret et al., 2016; Jones et al., 2017). There are two mechanisms so far described by which this may be achieved, activation of mGluR1 or M1Rs (Buchanan et al., 2010; Tigaret et al., 2016). In this study we show how each of these mechanisms may be engaged under distinct physiological conditions to enable gating of synaptic plasticity and therefore memory formation (Fig. 6). During active exploration when acetylcholine release is high (Teles-Grilo Ruivo et al., 2017) M1Rs can inhibit SK channels to open up a window for LTP induction by tightly correlated and theta/gamma entrained place-cell firing (Isaac et al., 2009). In contrast, during place-cell replay when acetylcholine release is low, coordinated high-frequency firing causes dendritic depolarization creating optimal conditions for activation of postsynaptic mGluR1 (Sylantyev et al., 2013; Tigaret et al., 2016) which again opens a window for LTP induction (Sadowski et al., 2016). The fact that SWRs are inhibited by acetylcholine release (Vandecasteele et al., 2014) implies that these two mechanisms are rarely coactive, however, there is also evidence that acetylcholine levels remain quite high during quiet wakefulness immediately following active exploration when SWRs are known to occur (Diba and Buzsáki, 2007; Teles-Grilo Ruivo et al., 2017) so there is potential for some overlap in the convergent mechanisms. Therefore, we propose that SK channels are a critical gate for LTP induction and that their inhibition by convergent metabotropic signaling pathways is required for LTP between place cells under varying physiological conditions. This framework suggests that the processes of memory formation and consolidation may be separately or jointly targeted by manipulation of M1Rs, mGluR1 or the convergent point of SK channels.

Muscarinic M1Rs activate a signaling pathway involving phospholipase C (PLC) and protein kinase C (PKC; Buchanan et al., 2010; but see Giessel and Sabatini, 2010) that reduces the sensitivity of SK channels to Ca^2+^ (Giessel and Sabatini, 2010) thereby inhibiting their ability to regulate NMDARs. The mechanism by which mGluR1 inhibits SK channels has not been fully tested but, given M1R and mGluR1 both signal via Gq-coupled pathways, it might be expected that mGluR1 inhibition of SK channels involves a similar signaling pathway to M1Rs. Indeed, M1Rs and Group 1 mGluRs have been shown to inhibit GIRK channels in CA1 pyramidal cells via similar but crucially non-identical pathways which converge on depletion of PIP2 (Sohn et al., 2007). In this instance, mGluR signaling is mediated by phospholipase A/arachidonic acid, whereas M1R signaling is via PLC/PKC. Other Gq-coupled receptors have also been shown to inhibit SK channels via distinct signaling pathways most notably noradrenergic receptors signaling via a casein kinase 2 pathway that reduces the Ca^2+^ sensitivity of SK channels (Maingret et al., 2008) and σ 1 receptors via an unknown pathway (Martina et al., 2007). Multiple neurotransmitter systems may therefore inhibit SK channels through a variety of intracellular signaling pathways but with a common target.

M1Rs and mGluR1 have been shown to act synergistically within CA1 pyramidal cells to regulate intrinsic excitability via regulation of R-type Ca^2+^ channels (Park and Spruston, 2012) and both M1Rs (Kim et al., 2002) and mGluR1 (Morishita et al., 1998) can facilitate the release of endocannabinoids from CA1 pyramidal cells to cause depolarization induced suppression of inhibition. Furthermore, M1Rs and Group 1 mGluRs act in tandem to promote non-Hebbian LTP at glutamatergic synapses on CA1 interneurons (Le Duigou et al., 2015). Interestingly, it has also been suggested that M1Rs and mGluR1 act selectively in functionally and anatomically distinct types of CA1 pyramidal cells to regulate plasticity of intrinsic excitability (Graves et al., 2012). Our data do not support such a cellular discretization of metabotropic control of spine Ca^2+^ dynamics and synaptic plasticity since the effects of M1Rs and mGluR1 are present consistently in all cells we recorded from. Furthermore, if such a discretized system existed for synaptic plasticity, it would imply that formation of memories occurs at synapses on one type of pyramidal cell and consolidation at synapses on another type.

Hebbian synaptic plasticity requires postsynaptic depolarization to remove the Mg^2+^ block of NMDARs. Synaptic depolarization is achieved in STDP with bAPs, but although these can produce a strong depolarization in proximal dendrites the depolarization in distal dendrites can be very limited (Callaway and Ross, 1995; Spruston et al., 1995). Synapses at a large electrotonic distance from the soma generally receive postsynaptic depolarization from dendritic spikes caused by suprathreshold summation of EPSPs mediated principally by NMDARs that likely require spatiotemporally clustered synaptic inputs (Stuart and Spruston, 2015). The suprathreshold summation of EPSPs caused by NMDAR activation is highly susceptible to regulation by potassium channels (Losonczy et al., 2008) including SK channels (Cai et al., 2004; Bock and Stuart, 2016). Therefore, it is predicted that synaptic plasticity induced by dendritic spikes, and indeed the initiation of dendritic spikes themselves, will also be gated by M1R or mGluR1 inhibition of SK channels. Given the emerging importance of dendritic spikes and resultant synaptic plasticity in generating feature selectivity in neurons (Takahashi et al., 2016), including place-field specificity in CA1 neurons (Sheffield and Dombeck, 2015; Bittner et al., 2017), this conveys SK channels with a critical role in neuronal information processing.

The primary function of the hippocampus is to form and consolidate new memories. In the rodent hippocampus this is best represented by spatial memory formed by binding together ensembles of place cells using synaptic plasticity (Harris et al., 2003; O'Neill et al., 2010). Memory formation is believed to be a two-stage process where ensembles are initially formed during active exploration and subsequently consolidated during off-line replay of the awake activity (O'Neill et al., 2010; Atherton et al., 2015). However, off-line replay occurs in different behavioral circumstances: The replayed place-cell spiking occurs on a greatly compressed timescale and during periods of transient high-frequency (100–200 Hz) activity in the local hippocampal network (SWRs) and found during periods of sleep or rest when neuromodulatory tone is low (Wilson and McNaughton, 1994; Lee and Wilson, 2002; Diba and Buzsáki, 2007; Teles-Grilo Ruivo et al., 2017). By comparison, place-cell activity during active exploration is entrained to theta (5–12 Hz) and gamma (40–120 Hz) frequency oscillations when neuromodulatory tone, in particular acetylcholine, is high (Dragoi et al., 2003; Dupret et al., 2010; Teles-Grilo Ruivo et al., 2017). In this study we reveal the mechanisms by which synaptic plasticity may be maintained under such varying physiological conditions and the critical role played by SK channels in gating synaptic plasticity.
