# Supplementary figures and images for: Convergent Metabotropic Signaling Pathways Inhibit SK Channels to Promote Synaptic Plasticity in the Hippocampus
Source: J Neurosci. 2018 Oct 24;38(43):9252–62. doi: 10.1523/JNEUROSCI.1160-18.2018 (PMC6199408; doi:10.1523/JNEUROSCI.1160-18.2018)

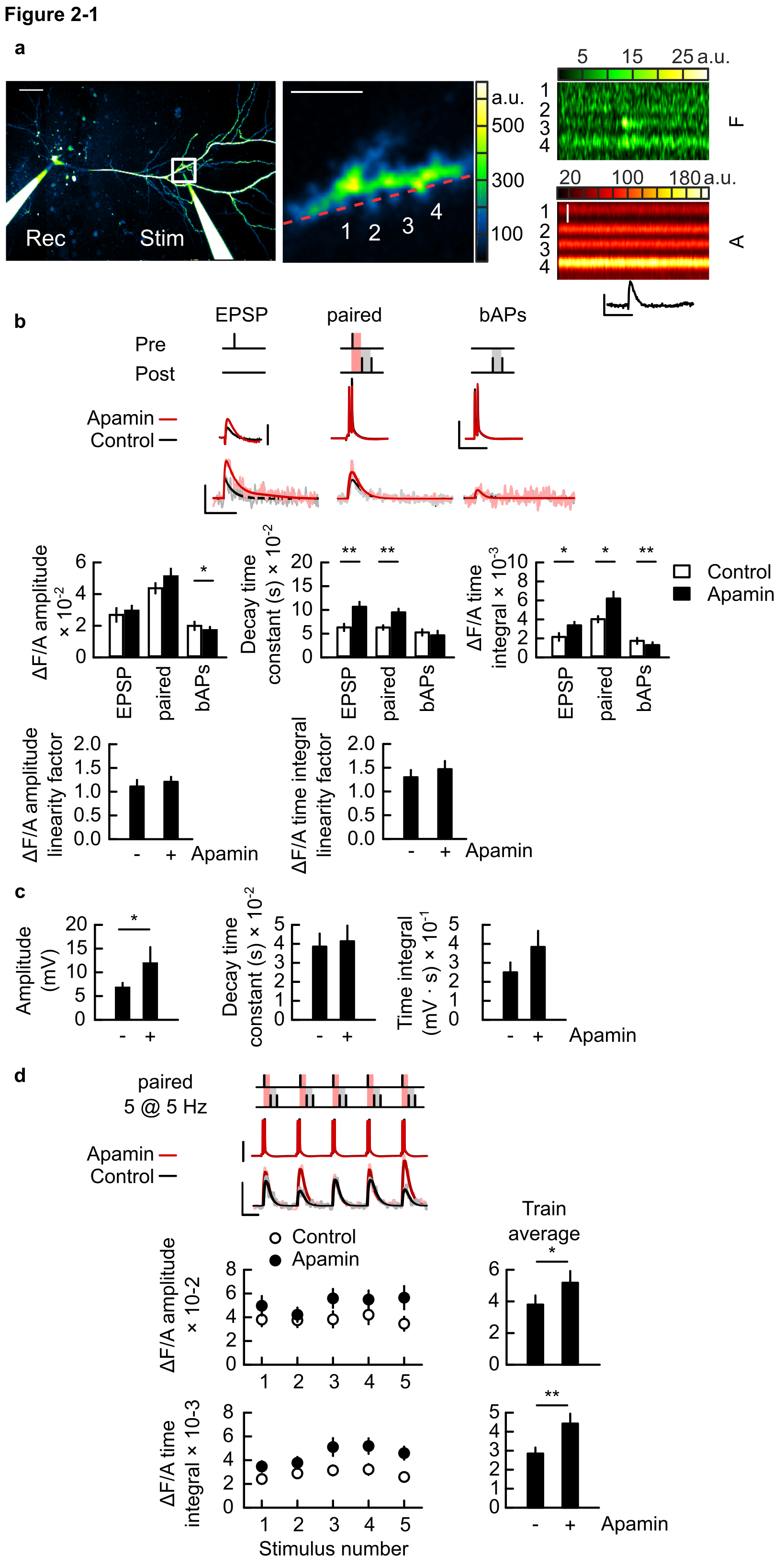

Supplement: Figure 2-1 [file zns999181172so1.tif]

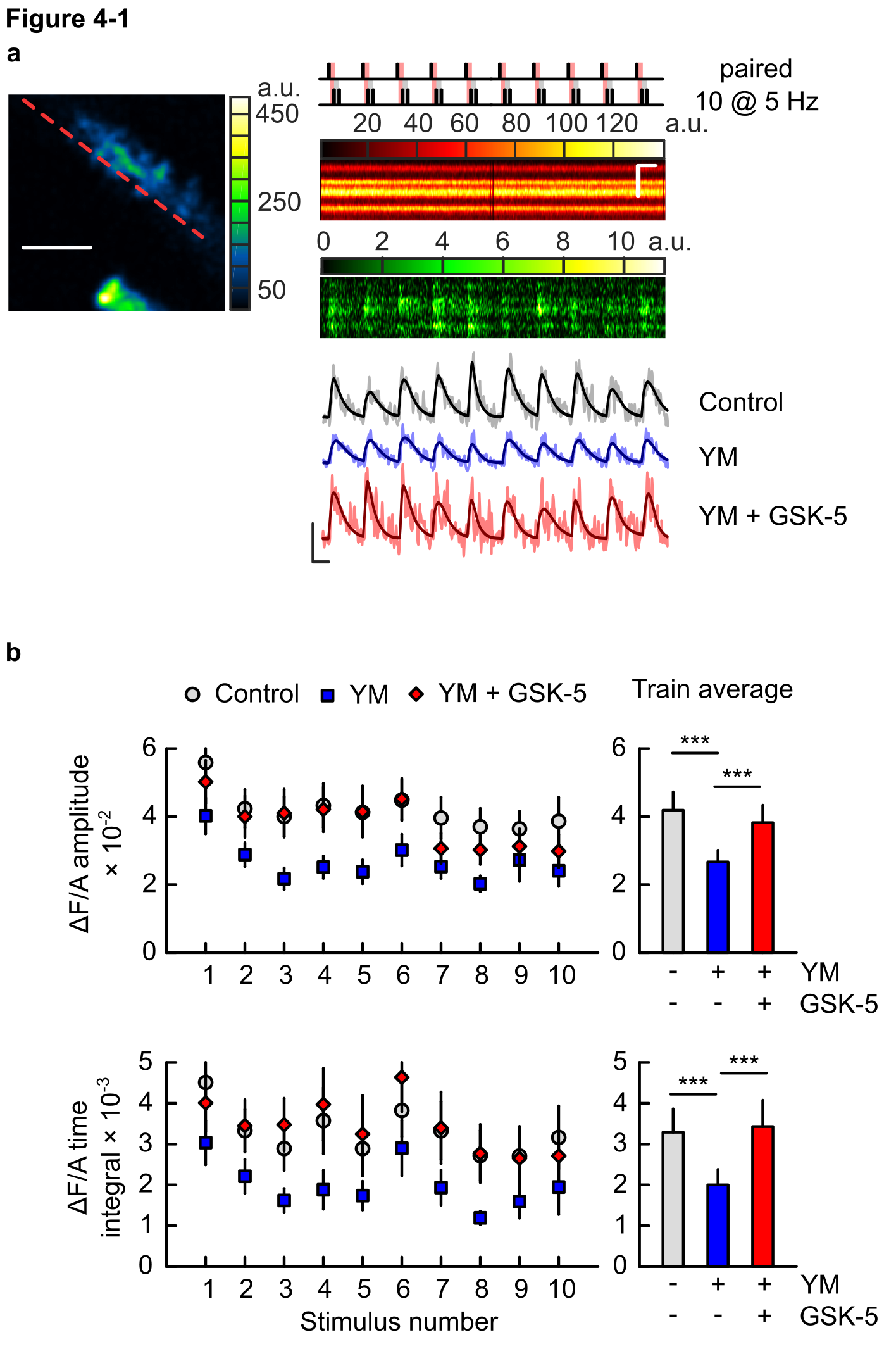

Supplement: Figure 3-1 [file zns999181172so2.tif]

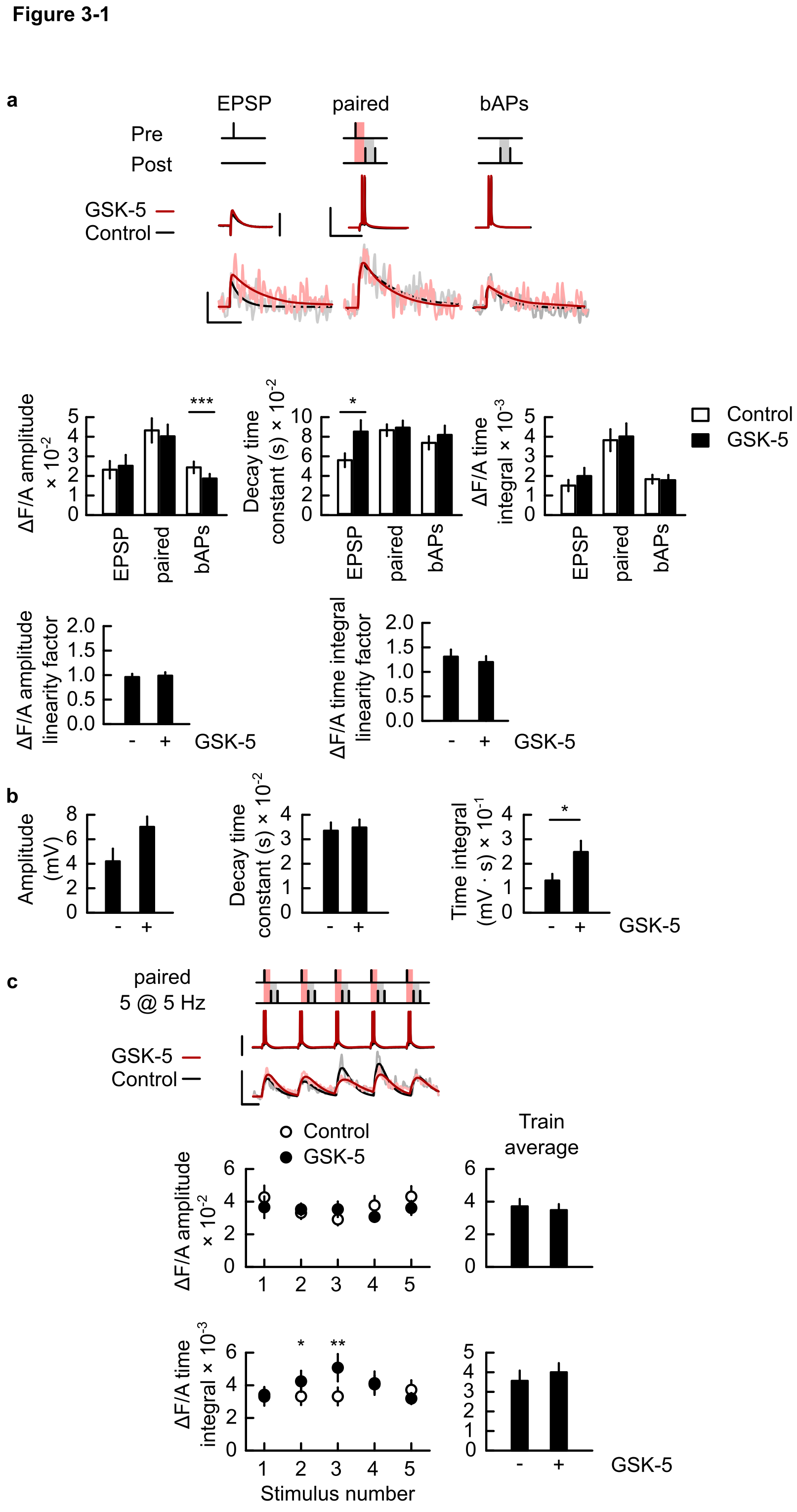

Supplement: Figure 4-1 [file zns999181172so3.tif]
